# Variant calling in genomics: A comparative performance analysis and decision guide

**DOI:** 10.1371/journal.pone.0339891

**Published:** 2026-02-05

**Authors:** Vera Pinto, Lisete Sousa, Carina Silva

**Affiliations:** 1 Departamento de Ciências Matemáticas (DCM)/Faculdade de Ciências, Universidade de Lisboa, Lisbon, Portugal; 2 Centro de Estatística e Aplicações (CEAUL)/Faculdade de Ciências, Universidade de Lisboa, Lisbon, Portugal; 3 ESSL – Escola Superior de Saúde de Lisboa, Instituto Politécnico de Lisboa, Lisbon, Portugal; Shaheed Rajaei Cardiovascular Medical and Research Center: Rajaie Cardiovascular Medical and Research Center, IRAN, ISLAMIC REPUBLIC OF

## Abstract

The accurate detection of genetic variants is critical for advancing genomics research and precision medicine. However, this task remains challenging due to pervasive sequencing errors and complex genomic regions. The choice of variant calling software significantly influences results, creating a need for clear, evidence-based guidance. This study aims to provide a performance evaluation and a clear, evidence-based guide for selecting variant callers by benchmarking seven widely used tools, GATK, FreeBayes, DeepVariant, Samtools, Strelka2, Octopus, and Varscan2, highlighting their algorithmic trade-offs. The well-characterized NA12878 genome from the Genome in a Bottle consortium was analyzed. High-coverage whole-genome sequencing data was processed with each variant caller, and the resulting variant calling files were benchmarked against a gold-standard reference. Performance was assessed using precision, recall, and F1-score on a chromosome 20 subset and on full whole-genome data. The analysis revealed that DeepVariant’s deep learning approach achieved the highest precision (0.7869) and F1-score (0.8754) on chromosome 20. For whole-genome analysis, Strelka2 excelled in precision (0.8326), while Octopus demonstrated superior recall (0.9838). FreeBayes exhibited high sensitivity but lower precision, underscoring a key trade-off. There is no universally superior variant caller; the optimal choice depends on the specific research objectives, whether prioritizing precision, recall, or computational efficiency. This study serves as a crucial evidence-based resource for researchers and clinicians, enabling informed tool selection.

## Introduction

Advances in high-throughput sequencing have significantly impacted genomic research and personalized medicine in recent years. The ability to decode genetic information has opened doors to a deeper understanding of genetic variation and its role in health and disease, particularly in genetic disorders. Variant Calling (VC), a crucial step following read alignment and quality filtering in the genomic data preprocessing pipeline, is essential for identifying deviations from a reference genome. Genomic deviations, known as variants, can be classified into several types, including single-nucleotide variations (SNVs), insertions, deletions, copy number variants (CNVs), and other structural variants (SVs). Variants are often grouped by size into SNVs, indels, and SVs (which encompass CNVs, duplications, and translocations). However, only a few variant callers are capable of detecting all three categories, as each requires distinct computational approaches [1]. Accurate and reliable variant calling is essential for understanding genomic mechanisms and identifying the genetic basis of disease [2]. In particular, detecting single-nucleotide polymorphisms (SNPs) and small insertions or deletions, collectively referred to as SNVs [3], provides valuable insights into disease mechanisms, potential therapeutic targets, and opportunities for personalized medicine.

Just as genetic variants exist in various forms, multiple methods and approaches have been developed for variant calling. Somatic and germline variant calling are two distinct processes, each with its own methodologies, applications, and downstream implications in the study of genetics and disease [4]. Somatic variant calling identifies mutations that occur in non-reproductive cells, typically acquired during a person’s lifetime, and are often observed in cancer. These mutations are not inherited and are distinguished from germline variants by comparison with a matched normal sample. In contrast, germline variant calling detects inherited variants present in every cell of an individual’s body, which can be passed to the next generation by analyzing a reference genome.

One challenge in somatic variant calling is disambiguating low-frequency variants from artifacts, which requires sensitive statistical modeling and advanced error correction technology [5]. For germline variant calling, a major challenge is distinguishing true genetic variants from sequencing errors and artifacts [6]. Despite significant advancements in sequencing technologies, errors such as base miscalls, low-quality reads, polymerase chain reaction (PCR) amplification artifacts, and mapping inaccuracies in repetitive or low-complexity regions can lead to false positive variant calls [7]. Moreover, analyzing genomic regions with high sequence similarity, such as duplicated genes or segmental duplications, adds further complexity to accurately identifying true variants [8].

To address these challenges, both statistical and heuristic approaches have been developed, each offering distinct advantages that align with the data and analysis objectives [9]. A statistical approach employs probabilistic models to evaluate variant likelihoods based on factors such as sequencing depth, base quality scores, and allele frequency [10]. These methods excel in handling complex scenarios, such as the low-frequency mutations common in somatic variant calling, and often incorporate machine learning and Bayesian models to reduce false positives caused by sequencing noise or artifacts [11–13]. In contrast, heuristic methods rely on predefined rules and thresholds to filter and call variants [14]. While heuristic methods are simpler, faster, and less computationally demanding, they are generally more effective for germline variant calling, where variants tend to be present at higher frequencies.

Despite all these efforts and the complex methodologies employed, accurately detecting genetic variants remains a challenging task due to factors such as sequencing errors [15], variable read coverage, and the intricate nature of certain genomic regions and variant types, as well as differences in the approaches of various variant callers [10,16,17].

The variation in detection results across different tools arises from the diverse error models, algorithmic assumptions, and methodological differences applied before and after variant calling [18,19]. Furthermore, sequencing errors and read alignment inaccuracies exacerbate these discrepancies, making precise variant detection a persistent challenge in genomics.

Despite the abundance of available tools, systematic performance assessments remain essential to help researchers select the most suitable option for their specific requirements [18,20]. The present study focuses on key evaluation metrics, precision, recall, and computational efficiency (running time and memory usage), to provide a valuable benchmark for the genomics community. These analyses aim to enhance understanding of current variant calling performance, inform future tool development, and ultimately promote more accurate and efficient variant detection in genetic research.

Under a standardized benchmarking study, the latest versions of established callers are compared with deep-learning and haplotype-based methods and the extent to which chr20 performance predicts whole-genome results is evaluated to determine whether subset-based tool selection is reliable.

Accordingly, the objectives of this study are as follows:

- To compare established and emerging variant callers under a standardized benchmarking workflow.
- To quantify the compute cost of each variant caller (CPU time, wall-clock time, memory, storage).
- To assess whether chr20 performance predicts whole-genome performance to justify subset-based tool selection.
- To translate benchmarking results into practical guidance for tool selection.

## Materials and methods

### Data

The dataset used in this study was a publicly available version sequenced with the TruSeq protocol on the Illumina HiSeq 2000 platform from BaseSpace Illumina (https://basespace.illumina.com/analyses/53043001/files?projectId=18065049). The whole-genome sequencing (WGS) data (approximately 50× mean coverage) for the sample NA12878 was obtained from a reference immortalized cell line. The WGS data was aligned using Isaac (version 04.16.09.24) according to the recommended Illumina pipeline; further details on the pipeline can be found in S1 File. All variant callers were applied to identical input data to ensure full comparability across tools. Specifically, each caller used the same Isaac-aligned BAM files as input, generated from the TruSeq-sequenced NA12878 dataset aligned to the hg38 reference genome. No additional preprocessing, realignment, or base quality score recalibration was applied beyond the Isaac pipeline defaults. This standardization ensured that any observed performance differences reflect the variant callers themselves rather than discrepancies in upstream data handling or filtering.

As a gold standard for validating the performance of the variant calling platform, the high-confidence reference variant calls for the 1000 Genome Project individual (sample NA12878) were used. These calls, published by the Genome in a Bottle (GIAB) consortium, were generated using the TruSeq panel from Illumina (https://ftp.ncbi.nlm.nih.gov/giab/ftp/data/NA12878/Nebraska_NA12878_HG001_TruSeq_Exome/). The available files include the BAM file (NIST-hg001-7001-ready.bam), the VCF file (NIST-hg001-7001-gatk-haplotype.vcf), and the BED file (TruSeq_exome_targeted_regions.hg19.bed), which covers 62,286,318 base pairs [21].

### Methods

#### Variant callers.

Seven software packages capable of single nucleotide variant (SNV) calling were evaluated: Samtools [22,23], GATK [14], FreeBayes [11], Strelka2 [6], DeepVariant [13], Octopus [24], and Varscan2 [25]. The selection of these seven variant callers was guided by their use in published studies, open-source availability, and suitability for short-read whole genome and exome sequencing data. They represent a diverse range of variant detection strategies, including heuristic methods, Bayesian inference, haplotype-based modeling, and modern machine learning techniques. This diversity ensures that the comparative evaluation reflects both well-established approaches and state-of-the-art developments in SNV calling, offering insights into their relative performance under consistent benchmarking conditions. All versions used were the latest available at the time of the study. To ensure full reproducibility of analyses, all software versions, command lines, and configurations used for variant calling and benchmarking are publicly available in our GitHub repository (GitHub repository).

Each tool accepts BAM files of aligned reads or pileups generated from BAM files (for example, those created by SAMtools’ mpileup function). For this study, all tools were configured to call variants in tumor-only mode (for somatic variant callers) or with default settings for germline calling, meaning that mutations were identified relative to the reference genome. Each software outputs a variant call format (VCF) file.

**Fig 1:**
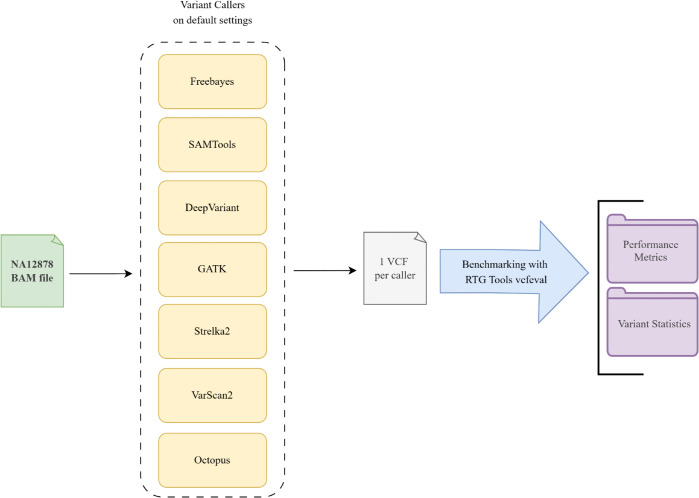
Workflow for comparative performance evaluation of variant callers. Schematic overview of the benchmarking methodology. Seven variant calling tools (FreeBayes, SAMTools, DeepVariant, GATK, Strelka2, VarScan2, and Octopus) were run on the same sequencing dataset using their default settings. Each tool generated a single Variant Call Format (VCF) file output. These VCF files were then evaluated against a high-confidence gold standard variant set using RTG Tools’ vcfeval function. This benchmarking process produced two primary categories of results: Performance Metrics (Precision, Recall, and F1-score) and detailed Variant Statistics (e.g., Ti/Tv ratio, Het/Hom ratio, indel counts).

Each variant caller comes with preset, yet adjustable, parameters such as minimum variant frequency, minimum coverage, minimum base quality score, and minimum mapping quality score. For analysis, the default recommended settings for each tool were used as showed in Fig 1.

#### Analysis strategy.

The analysis was performed twice to ensure robustness in variant calling: first on a subset of the data comprising only chromosome 20, and then on the whole-genome sequence. This dual approach allowed a comparison of each tool’s performance and computational efficiency, both within a limited genomic region and across the entire genome. Analyzing chromosome 20 provided a quick assessment of each tool’s accuracy in a smaller region, while whole-genome analysis evaluated scalability and overall SNV detection precision [10,12].

All steps of the analysis were run in parallel, either using built-in multi-threading options (when supported by the tool) or by running multiple instances of each tool in parallel on smaller data chunks.

Due to computational constraints and available hardware, DeepVariant and Varscan2 were unable to complete the runs on the WGS dataset, and Strelka2 was unable to complete the run on the chromosome 20 subset due to difficulties in analyzing segmented genomes.

#### Performance assessment.

For performance evaluation, the following standard metrics were calculated. The **precision** is defined as the ratio of true positives (*TP*) to the sum of true positives and false positives (*FP*). That is,

$$Precision=\frac{TP}{TP+FP},$$ (1)

where *TP* + *FP* are counts of positives detections.

Similarly, the **recall** (or sensitivity) is given as the ratio of true positives to the sum of true positives and false negatives (*FN*):

$$Recall=\frac{TP}{TP+FN},$$ (2)

with *TP* + *FN* are the total number of positives in the data set.

Finally, the **F1-score** is computed as the harmonic mean of precision and recall:

$$F1=2\;\frac{Precision\times Recall}{Precision+Recall},$$ (3)

which balances the trade-off between precision and recall.

For the NA12878 cell line, the gold-standard variant calls from [21] were used as the reference for true SNVs. The identified SNVs were compared against this reference using RTG Tools’ vcfeval function [26] and VariantEval from GATK. The vcfeval tool facilitates a detailed comparison of VCFs by evaluating the F1-score (see Eq 3) based on counts of true positives (TP), false negatives (FN), and false positives (FP). A variant that matches one variant from the gold standard is classified as a TP; if a variant within the expected genomic intervals is not detected, it is marked as an FN; and any variant detected within the regions but not present in the gold standard is considered an FP. Performance metrics such as precision (Eq 1), recall (Eq 2) and a F1-score (Eq 3), were computed to assess overall performance.

This approach provides a robust means of evaluating variant calling tools, as RTG Tools [26] ensures an accurate comparison of the variant caller outputs with a trusted reference dataset.

#### Correlation analysis between Chr20 and WGS.

To evaluate whether chromosome 20 benchmarking results predict genome-wide behavior, we computed Spearman’s rank correlation coefficients (*ρ*) and their two-tailed *p*-values between chr20 and WGS values for precision, recall, and F1-score across the four variant callers that completed both analyses (GATK, Octopus, FreeBayes, SAMtools). For each metric, chr20 (*x*) and WGS (*y*) values were ranked and Spearman’s *ρ* was obtained with scipy.stats.spearmanr (Python v3.10; SciPy v1.12.0).

All calculations were performed in Python (v3.10) using the scipy.stats.spearmanr function (SciPy v1.12.0). The resulting Spearman rank coefficients (*ρ*) and two-tailed *p*-values were reported for each metric. Because only four callers overlapped across scales (*n* = 4), *p*-values were interpreted with caution, emphasizing the effect sizes (*ρ*) and the consistency of direction across metrics.

#### Computational efficiency

In addition to variant detection performance, computational efficiency was assessed based on memory usage and runtime required for each tool.

## Results

In this section, the key findings from the analysis are presented in relation to summary statistics of each variant caller and its performance metrics. The results have been organized into four key areas: (i) variant calling statistics computed on the NA12878 subset for chromosome 20, (ii) variant calling statistics computed on NA12878 whole-genome sequencing (WGS) data, (iii) performance metrics on the NA12878 subset for chromosome 20, and (iv) performance metrics on WGS data.

### Characteristics on subset for chromosome 20

According to S1 Table, which summarizes the output characteristics of each variant caller on the chromosome 20 subset (chr20), several indicators were used to assess the behavior of each tool. These include the number of passed and failed variant filters, total counts of SNPs, insertions, and deletions, as well as ratios such as transition transversion (Ti/Tv), heterozygous/homozygous (Het/Hom), and insertion/deletion (In/Del). These metrics reflect how stringent or permissive each caller is, as well as the quality and balance of variant calls.

#### Filtering behavior and total variant calls.

DeepVariant stands out as the most stringent tool, with 59,970 variants failing the filters. This suggests that DeepVariant applies strong quality thresholds to prioritize high-confidence calls and reduce false positives. Octopus and Varscan2 also apply notable filtering (9,080 and 2,896 failed variants, respectively), albeit less stringently. In contrast, FreeBayes and Samtools report zero failed filters, indicating highly permissive behavior. FreeBayes, in particular, detects the highest number of variants (328,360), while GATK and Samtools present a balanced approach with 123,220 and 116,952 passed variants, respectively.

#### SNP detection.

GATK reports the highest number of SNPs (103,825), consistent with its known strength in identifying single nucleotide variants. Samtools and FreeBayes also detect high SNP counts (101,639 and 100,911, respectively), suggesting a more liberal calling approach. DeepVariant detects the fewest SNPs (85,840), likely due to its prioritization of precision over recall. Octopus and Varscan2 fall in between, with 89,832 and 93,615 SNPs, respectively.

#### Indel detection.

Regarding indels (insertions + deletions), Octopus detects the highest number (19,605), with a balanced distribution between insertions (10,323) and deletions (9,282). FreeBayes and Samtools follow, with 16,311 and 15,146 indels, respectively. Varscan2 reports lower indel counts (13,247), while GATK (275) and DeepVariant (346) detect very few indels, reflecting their stricter filtering behavior.

#### Transition/Transversion (Ti/Tv) ratio.

The Ti/Tv ratio is a key SNP quality indicator. DeepVariant achieves the expected human genome value of 2.00, suggesting high-quality SNP calls. Octopus and Varscan2 also present high Ti/Tv ratios (2.07 and 2.03), indicating well-calibrated SNP calling. GATK (1.92) and Samtools (1.93) follow closely, while FreeBayes shows the lowest ratio (1.88), which may suggest a slightly increased rate of false positive transversions.

#### Het/Hom ratios.

The total Het/Hom ratio indicates the balance between heterozygous and homozygous variant detection. GATK shows the highest ratio (2.50), suggesting high sensitivity to heterozygous variants. FreeBayes (2.39), Varscan2 (2.37), and Octopus (2.07) also display strong heterozygous detection. Samtools has a lower ratio (2.13), while DeepVariant (1.77) appears more conservative, potentially reducing false heterozygous calls.

#### Insertion/Deletion (In/Del) ratio.

A balanced In/Del ratio near 1.0 is desirable. Octopus (1.11), DeepVariant (0.97), GATK (0.96), and Varscan2 (0.98) maintain well-balanced insertions and deletions. FreeBayes is slightly deletion-biased (0.89), and Samtools shows a mild insertion bias (1.06). These ratios suggest that, except for FreeBayes, all tools handle both indel types with reasonable balance.

### Characteristics on WGS

According to S2 Table, which summarizes the variant calling statistics on NA12878 whole-genome sequencing (WGS) data, the tools show substantial differences across multiple performance indicators. These include the number of passed variants, SNP and indel counts, Ti/Tv ratios, Het/Hom ratios, and the balance between insertions and deletions. Each metric provides insight into the tool’s sensitivity, precision, and internal filtering strategy.

#### Filtering behavior and total variant calls.

FreeBayes reports the highest number of passed variants (12,560,020), substantially more than all other tools, suggesting a highly permissive approach that prioritizes recall by capturing a broader spectrum of potential variants, albeit with an increased risk of false positives. In contrast, GATK, Strelka2, Samtools, and Octopus each report approximately 4.5 to 4.9 million passed variants, indicating the use of stricter filtering thresholds that likely enhance precision.

#### SNP detection.

Samtools detected the highest number of SNPs (4,119,113), closely followed by GATK and FreeBayes. While Strelka2 and Octopus reported slightly fewer SNPs (3,796,849 and 3,856,679, respectively), this may reflect stricter filtering that improves call confidence. FreeBayes and GATK presented similar SNP counts, highlighting their sensitivity; however, FreeBayes’ liberal calling strategy may introduce more false positives, whereas GATK is designed to maintain a balance between recall and precision.

#### Indel detection.

FreeBayes reported the highest number of indels (103,179), far exceeding those detected by the other tools. GATK, Strelka2, and Samtools detected around 13,000 indels each, suggesting that these tools share similar stringency levels, potentially due to local realignment strategies that improve indel accuracy. Octopus reported the fewest indels (3,396), reflecting a more conservative and precision-focused approach.

#### Transition/Transversion (Ti/Tv) Ratio.

Ti/Tv ratios were highest for Strelka2 and Octopus (both 2.02), indicating high-quality SNP calls with fewer false positive transversions. GATK and FreeBayes both had Ti/Tv ratios of 1.95, while Samtools had a slightly lower ratio (1.93), suggesting minor variations in stringency among the tools.

#### Het/Hom Ratios.

The total Het/Hom ratios were highest for FreeBayes and GATK (both 1.71), indicating a strong ability to detect heterozygous variants. Octopus and Strelka2 showed slightly lower ratios (1.60 and 1.57, respectively), suggesting a more conservative profile. SNP-specific Het/Hom ratios followed a similar trend, with FreeBayes and GATK favoring heterozygous detection more than Octopus, Samtools, and Strelka2.

#### Insertion/Deletion (In/Del) Ratio.

Most tools showed balanced insertion-to-deletion ratios. Samtools had the most balanced profile (1.01), while GATK (0.95), Strelka2 (0.97), and Octopus (1.09) were close to 1.0, reflecting consistent handling of both variant types. FreeBayes was slightly biased toward deletions (0.88), which may affect downstream interpretation depending on the biological context.

### Performance metrics for chromosome 20

**Fig 2:**
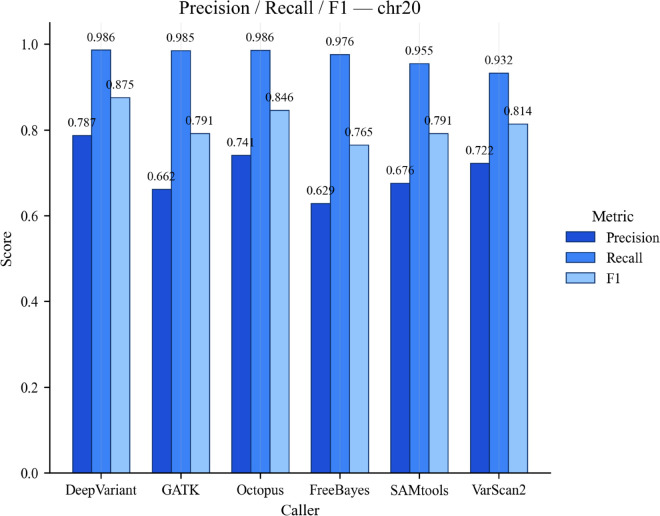
Performance metrics for variant callers on chromosome 20. Comparison of precision, recall, and F1-score across seven variant calling tools evaluated on the NA12878 chromosome 20 subset. All metrics were calculated using RTG Tools’ vcfeval against the Genome in a Bottle consortium gold standard reference.

Based on the data presented in S3 Table and in Fig 2, DeepVariant demonstrates the highest precision precision (0.7869), indicating a low rate of false positives among its variant calls, likely due to its deep learning-based approach. Octopus and Varscan2 also perform well, with precision values of 0.7412 and 0.7220, respectively, suggesting that they apply rigorous filtering criteria. Samtools and GATK exhibit moderate precision at 0.6761 and 0.6617, reflecting a balance between precision and recall. FreeBayes, with the lowest precision (0.6286), may be more prone to false positives, particularly in single-sample or low-coverage regions.

Recall remains consistently high across all tools, highlighting their effectiveness in identifying true variants. DeepVariant achieves the highest recall (0.9864), closely followed by Octopus (0.9862) and GATK (0.9847), indicating their ability to capture a substantial number of true variants. Samtools and FreeBayes show slightly lower recall (0.9545 and 0.9758, respectively), while Varscan2, with a recall of 0.9322, is comparatively less sensitive. The high recall for GATK is likely due to techniques like local haplotype reassembly [14], and DeepVariant’s high recall is primarily attributed to its advanced machine learning framework, robust handling of sequencing noise, and implicit modeling of variant evidence [13].

In terms of the F1-score, which balances precision and recall, DeepVariant leads with an F1-score of 0.8754, reflecting its overall effectiveness. Octopus follows with an F1-score of 0.8463, while Varscan2 achieves an F1-score of 0.8137. Samtools and GATK both have an F1-score of 0.7915, and FreeBayes has the lowest F1-score (0.7646), likely due to its lower precision despite high recall.

### Performance metrics for whole-genome sequencing data

**Fig 3:**
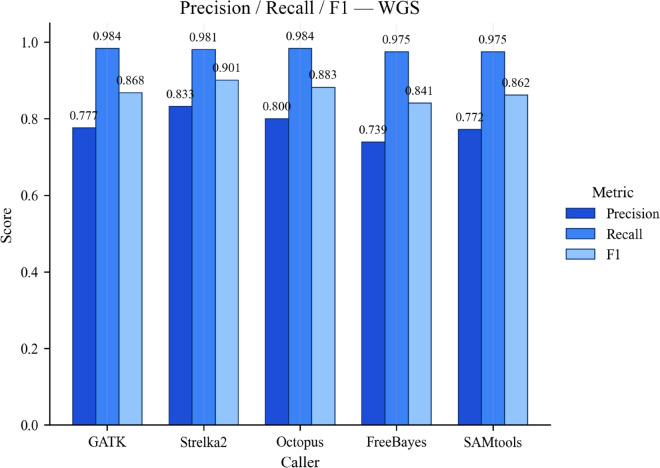
Performance metrics for variant callers on whole-genome (WGS). Comparison of precision, recall, and F1-score across five variant calling tools (GATK, Strelka2, Octopus, FreeBayes, SAMtools) evaluated on the NA12878 whole-genome dataset. All metrics were computed with RTG Tools’ vcfeval against the Genome in a Bottle (GIAB) truth set.

According to S4 Table and Fig 3, Strelka2 achieves the highest precision (0.8326), followed by Octopus (0.8005) and GATK (0.7766). Samtools and FreeBayes show slightly lower precision values, at 0.7722 and 0.7394, respectively. The high precision of GATK and Octopus, improved from the chr20 subset, may be attributed to enhanced local realignment and optimized error models for whole-genome analysis. GATK’s consistent precision highlights its robust reassembly algorithm, while FreeBayes’ lower precision suggests a higher rate of false positives at the genome-wide scale.

All tools exhibit high recall, with Octopus achieving the highest recall (0.9838), closely followed by GATK (0.9837) and Strelka2 (0.9813). Samtools (0.9754) and FreeBayes (0.9748) also perform well, indicating strong sensitivity. In terms of the F1-score, Strelka2 leads with 0.9009, demonstrating its overall strength in genome-wide analysis. Octopus, with an F1-score of 0.8827, also shows a balanced approach. GATK and Samtools achieve F1-scores of 0.868 and 0.862, respectively, while FreeBayes has the lowest F1-score (0.8409), primarily due to its lower precision despite high recall.

In summary, Strelka2’s combination of high precision and recall positions it as the best overall performer for genome-wide analysis. GATK and Octopus are also strong contenders, with Octopus particularly excelling in recall. FreeBayes, while maintaining robust recall, suffers from lower precision, affecting its overall F1-score.

**Fig 4:**
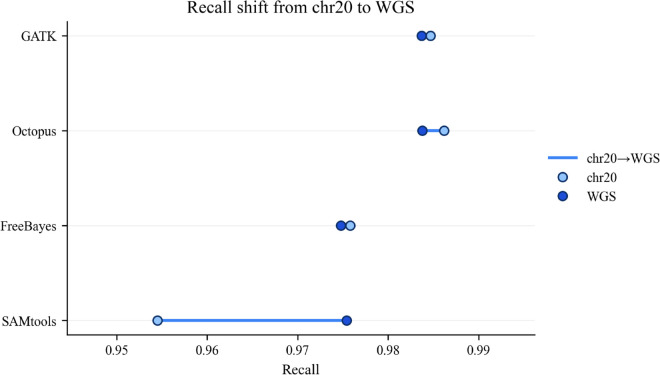
Stability of recall from chromosome 20 to whole-genome sequencing. Comparison of recall metrics for four variant callers (GATK, Octopus, FreeBayes, SAMtools) between the chromosome 20 subset and the whole-genome (WGS) dataset. Recall remained highly stable across sequencing scales for most tools, with GATK, Octopus, and FreeBayes showing negligible change (Δ<±0.002). SAMtools was the only caller to show improvement, increasing from 0.955 on chr20 to 0.975 on WGS (Δ=+0.020). The high consistency in recall suggests that the fundamental sensitivity of these variant callers is not dependent on the scale of the input data. Metrics were computed with RTG Tools’ vcfeval against the Genome in a Bottle (GIAB) truth set.

**Fig 5:**
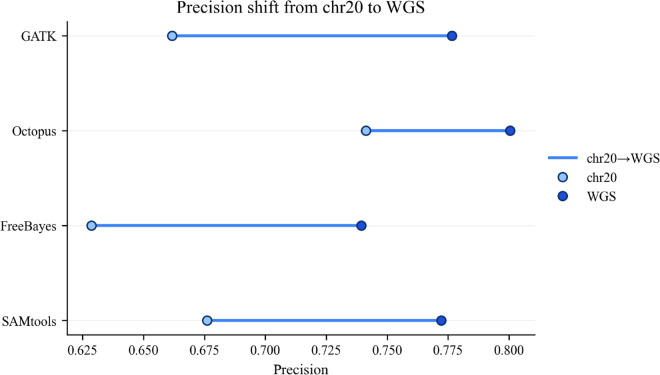
Change in precision from chromosome 20 to whole-genome sequencing. Comparison of precision metrics for four variant callers (GATK, Octopus, FreeBayes, SAMtools) between the chromosome 20 subset and the whole-genome (WGS) dataset. All tools showed increase in precision on WGS data. GATK exhibited the largest improvement (from 0.662 to 0.777, Δ=+0.115), followed closely by FreeBayes (Δ=+0.110). Octopus, which had the highest precision on chr20 (0.741), maintained the highest absolute precision on WGS (0.801) with a gain of +0.060. The precision shift suggests that a whole-genome context provides enhanced statistical power for filtering false positives. Metrics were computed with RTG Tools’ vcfeval against the Genome in a Bottle (GIAB) truth set.

**Fig 6:**
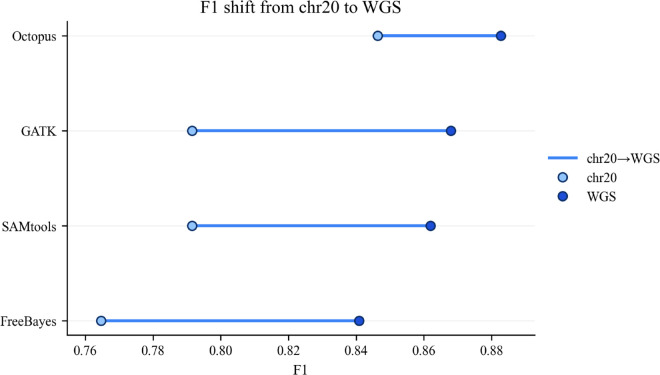
Change in F1-score from chromosome 20 to whole-genome sequencing. Comparison of F1-scores for four variant callers (Octopus, GATK, SAMtools, FreeBayes) between the chromosome 20 subset and the whole-genome (WGS) dataset. All tools showed improved F1-scores on WGS data, driven primarily by gains in precision. GATK exhibited the largest gain (Δ=+0.078), improving from 0.791 to 0.869. FreeBayes and SAMtools showed similar improvements (Δ=+0.076 and Δ=+0.070, respectively). Octopus, which started with the highest F1-score on chr20 (0.846), showed a moderate gain (Δ=+0.037) but achieved the highest final F1-score of 0.883 on WGS. Metrics were computed with RTG Tools’ vcfeval against the Genome in a Bottle (GIAB) truth set.

The performance of the four variant callers (GATK, Octopus, FreeBayes, and SAMtools) was assessed on both a chromosome 20 (chr20) subset and a whole-genome sequencing (WGS) dataset. The changes in precision, recall, and F1-score between these two scales are summarized in Figs 4, 5 and 6.

Recall remained stable across sequencing scales as seen in Fig 4. Recall values were highly consistent between the chr20 and WGS analyses for most tools. GATK, Octopus, and FreeBayes showed negligible change (< ±0.002), indicating their sensitivity was unaffected by the scale of the input data. SAMtools was the only caller to show a noticeable increase in recall (+0.020), improving from 0.955 on chr20 to 0.975 on WGS.

Precision in Fig 5 improved for all callers on WGS data. All four tools demonstrated a substantial increase in precision when moving from the chr20 subset to the full WGS analysis. GATK showed the largest absolute gain (+0.115), rising from a precision of 0.662 on chr20 to 0.777 on WGS. FreeBayes also showed a major improvement (+0.110), followed by SAMtools (+0.096) and Octopus (+0.060). Despite these gains, Octopus maintained the highest absolute precision on both datasets.

The F1-score in Fig 6, which balances precision and recall, increased for all four tools, driven primarily by the improvements in precision. GATK exhibited the largest gain in F1-score (+0.078). FreeBayes and SAMtools showed similar substantial improvements (+0.076 and +0.070, respectively). Octopus, which started with the highest F1-score on chr20, showed a more moderate gain (+0.037) but achieved the highest final F1-score of 0.883 on the WGS data.

To assess whether results obtained on chromosome 20 predict genome-wide behavior, we computed *Spearman’s* rank correlation coefficients (*ρ*) between chr20 and WGS values for precision, recall, and F1-score across the four callers evaluated on both scales (GATK, Octopus, FreeBayes, SAMtools).

Positive, high monotonic associations were observed between chr20 and WGS: *r*_*s*_ = 0.800 for precision, *r*_*s*_ = 0.800 for recall, and *r*_*s*_ = 0.949 for F_1_-score. Given the small number of paired observations (*n* = 4), hypothesis testing was not performed $H_{0}\;:\rho =0$ and therefore do not report *p*-values; interpretation focuses on the magnitude and direction of *r*_*s*_. Overall, these associations indicate consistent performance patterns between chr20 and WGS, supporting chr20 benchmarking as a useful, lower-cost proxy for genome-wide evaluations.

### Computer efficiency

The computational efficiency of variant callers varies significantly in terms of runtime and memory usage, depending on their underlying algorithms and the size of the input dataset. To evaluate efficiency, runtimes were analyzed on the chromosome 20 subset and on WGS data, along with memory requirements, (a information summary is given in Table 1). Samtools demonstrated short runtimes and low memory usage, making it highly efficient though less precise in complex regions. In contrast, DeepVariant and Octopus exhibited longer runtimes and higher memory demands due to their advanced modeling and computational complexity, which are offset by their superior precision and F1-scores. Tools like FreeBayes and Strelka2 balanced moderate runtimes with reasonable memory usage, offering a trade-off between efficiency and accuracy. Finally, Varscan2, while slower, showed low memory requirements, making it suitable for resource-constrained environments. The runtimes assume a typical machine with 12 CPU cores and 16 GB RAM. Note that tools like DeepVariant benefit from GPU acceleration, which can significantly reduce runtimes for large datasets.

**Table 1:** Computational efficiency of variant calling tools.

Caller	Chr20 Runtime	WGS Runtime	Memory	Efficiency
Strelka2	-	Moderate (5-8 h)	Moderate	Efficient, good for somatic variants
FreeBayes	Moderate (30min-2h)	Moderate (5-8 h)	Moderate	Good speed-memory balance
Samtools	Short (<30min)	Moderate (2-4 h)	Low	Lightweight, efficient
GATK	Moderate (30min-2h)	Long (10-15 h)	High	Accurate but intensive
Octopus	Long (2-5 h)	Very Long (>20 h)	High	Accurate but slow
VarScan2	Very Long (>5 h)	-	Low	Slow, low memory
DeepVariant	Very Long (>10 h)	-	Very High	Precise, needs GPU

Note: Runtime estimates based on NA12878 dataset using 12 CPU cores and 16 GB RAM. Tools marked ’-’ were not evaluated on that specific dataset.

To contextualize these findings for large-scale analyses, it was estimated approximate runtime scaling for cohort studies based on observed single-sample runtimes (Table 1). Assuming near-linear scaling under identical hardware (12 CPU cores, 16 GB RAM), processing 100 whole genomes would require approximately 200–400 CPU hours for SAMtools and Strelka2, compared to >1,000 CPU hours for GATK HaplotypeCaller and > 2,000 CPU hours for Octopus or DeepVariant without GPU acceleration. Published benchmarks support these relative magnitudes, reporting sub-hour runtimes for Strelka2 [6], multi-hour CPU runtimes for GATK [14], and substantially higher computational costs for Octopus [24] and DeepVariant [12]. Both Strelka2 and DeepVariant support distributed and GPU-optimized workflows that can reduce total wall-clock time by one order of magnitude in multi-node or cloud environments. These findings highlight the practical trade-off between accuracy and computational cost in large cohort studies, where precision-focused models may demand greater resources but deliver higher confidence in variant calls.

## Discussion

Variant calling performance is closely tied to the statistical methodologies employed, as evidenced by metrics like precision, recall, and F1-score across chr20 and WGS datasets (Tables S3 and S4). The algorithms analyzed here, FreeBayes, GATK, DeepVariant, Samtools, Octopus, Strelka2, and Varscan2, use distinct approaches that influence their detection capabilities (see summarized information in Table 2), particularly with respect to their handling of false positives and true positives. This discussion will highlight how each algorithm’s statistical framework influences these performance results.

**Table 2:** Characterization of variant callers.

Comprehensive Characterization of Variant Calling Tools				
**Variant Caller**	**Type**	**Methodology**	**Advantages**	**Limitations**
Samtools	Germline	Bayesian model	Efficient, lightweight	Lower recall in complex regions
GATK	Germline	Bayesian framework	High precision	High memory demand
FreeBayes	Both	Haplotype-aware	Multi-sample support	Lower precision single-sample
Strelka2	Somatic	ML filtering	High somatic recall	High false positives
DeepVariant	Germline	Deep learning	Accurate across technologies	High computational cost
Octopus	Both	Bayesian with HMM	High precision	High resource usage
Varscan2	Somatic	Heuristic methods	High somatic recall	Lower indel precision

Comparison of variant calling tools including their primary application type, statistical methodology, advantages, and limitations.

### Bayesian approaches and haplotype-based methods

Algorithms like GATK, FreeBayes, and Octopus leverage Bayesian inference and haplotype-based approaches to achieve high recall rates, which makes them highly sensitive to detecting true genetic variants. These tools use prior probability distributions and complex statistical models to distinguish sequencing errors from genuine variants. By incorporating both data likelihoods and prior probabilities, Bayesian methods enable these algorithms to make informed variant-calling decisions, ultimately increasing their overall performance and precision.

#### GATK—HaplotypeCaller.

GATK’s HaplotypeCaller is one of the most widely used variant callers in genomics and employs a sophisticated Bayesian framework with graph-based local haplotype assembly to enhance variant detection [14]. The HaplotypeCaller algorithm begins by examining each locus in the genome and then locally assembles overlapping reads into haplotype graphs. This local assembly step enables GATK to detect variants by reconstructing small portions of the genome directly from the reads, which is particularly valuable in complex regions where alignment errors are common. The algorithm computes genotype likelihoods for each haplotype, comparing the observed reads to possible genotype combinations (homozygous reference, heterozygous, homozygous alternate) [14]. It applies a Bayesian model to calculate posterior probabilities, which combines prior information (such as known population allele frequencies) with the likelihood of the observed data given each possible genotype. By maximizing this posterior probability, GATK can select the most likely genotype for each position [14]. GATK’s haplotype-based approach significantly improves its ability to accurately call SNPs, small insertions, and deletions, especially in challenging genomic regions with repetitive sequences or complex variant structures. This approach contributes to its high recall rates (0.9847 in chr20 and 0.9837 in WGS) while maintaining a balanced precision (0.6617 in chr20 and 0.7766 in WGS). The use of local haplotype assembly enables GATK to balance sensitivity and precision effectively, minimizing the proportion of false positives among its predictions and consequently, increasing the number of true positives by recalibrating and realigning reads, thereby improving the confidence in variant calls.

#### FreeBayes.

FreeBayes uses a haplotype-aware Bayesian model that allows it to detect a wide variety of variant types, including SNPs, indels, and multi-allelic loci [11]. Unlike GATK, which performs local reassembly for each variant, FreeBayes operates directly on the alignment data, looking for mismatches between the reads and the reference genome. FreeBayes is particularly well-suited for multi-sample and polyploid data due to its haplotype-aware approach, which allows it to call variants across multiple samples simultaneously and estimate genotype probabilities based on observed allele frequencies within the population [11]. The Bayesian framework in FreeBayes incorporates prior probabilities based on known allele frequencies and uses a likelihood model that assesses the probability of observing the sequencing data given each possible genotype [11]. This model takes into account sequencing errors, mapping quality, and read depth. However, FreeBayes generally applies less restrictive priors than GATK, leading to a more permissive calling strategy. This permissiveness results in high recall values (0.9758 for chr20 and 0.9748 for WGS), indicating that FreeBayes is highly sensitive and capable of detecting a large number of true variants. However, FreeBayes’s less conservative Bayesian priors contribute to a lower precision (0.6286 for chr20 and 0.7394 for WGS). The algorithm’s tendency to retain variants with lower support makes it more susceptible to calling false positives, especially in single-sample or low-coverage settings where distinguishing real variants from sequencing artifacts becomes challenging. In these cases, FreeBayes may struggle to filter out sequencing errors, resulting in a higher rate of over-calling compared to more restrictive algorithms like GATK. FreeBayes applies less restrictive priors to maximize flexibility and adaptability across a variety of sequencing scenarios, including complex ploidy and heterogeneous populations. In contrast, GATK uses stricter priors to optimize for high-confidence variant calling, particularly in well-characterized and high-quality datasets like diploid human genomes. This difference reflects the contrasting goals of the two tools: FreeBayes prioritizes sensitivity and adaptability, while GATK emphasizes precision and recall.

#### Octopus.

Octopus is another advanced variant caller that combines Bayesian inference with a hidden Markov model (HMM) for haplotype-based variant calling [24]. Octopus begins by building candidate haplotypes from sequencing reads, then uses an HMM to compute the likelihood of observing each read given each candidate haplotype. The HMM structure allows Octopus to align reads to haplotypes dynamically, adjusting for potential alignment errors and improving its precision in detecting true variants. This haplotype-aware Bayesian framework in Octopus provides high precision and recall by focusing on the likelihood of specific haplotypes rather than individual bases. The algorithm calculates posterior probabilities for each possible genotype by combining the prior probability of the haplotype with the likelihood of the observed data. This Bayesian-HMM approach is particularly advantageous in regions with complex structural variations or high levels of polymorphism, where it helps to reduce false positives by assigning higher confidence to more probable haplotypes [24]. In WGS data, Octopus achieves a high recall (0.9838), demonstrating its sensitivity in detecting true variants even in challenging genomic contexts. This high recall, coupled with its precision (0.7412 in chr20 and 0.8005 in WGS), suggests that Octopus has a balanced approach to variant calling that reduces false positives more effectively than FreeBayes while maintaining high recall. However, Octopus may still exhibit a higher rate of over-calling compared to stricter tools like Strelka2, particularly in simpler regions where the complexity of its HMM model may not be as beneficial. For instance, in cases of low data quality or insufficient sequencing coverage, the full potential of haplotype modeling cannot be effectively realized, as the limited evidence may hinder the accurate reconstruction of haplotypes. Additionally, for straightforward variants, such as SNPs or small indels that are well-supported by sequencing reads, simpler single-variant models can achieve comparable precision without the need for the computational overhead associated with HMM-based approaches. Moreover, in contexts where computational efficiency is a priority, such as WGS studies or high-throughput workflows, the increased complexity of the HMM model may not justify the marginal improvements in precision it provides. This is particularly relevant in repetitive genomic regions or those with low complexity, where the ambiguity in mapping sequencing reads reduces the reliability of haplotype modeling.

In summary, GATK, FreeBayes, and Octopus all use Bayesian inference to integrate prior knowledge with observed data, enhancing their recall. GATK’s local haplotype assembly enables high precision in complex regions, balancing precision and recall by minimizing false positives through realignment and recalibration. FreeBayes, with its permissive priors, achieves high recall but is more prone to false positives, particularly in low-coverage or single-sample data. Octopus combines Bayesian inference with an HMM-based haplotype model, providing both high recall and improved precision in complex regions by dynamically aligning reads to haplotypes. These methodological distinctions contribute to each algorithm’s performance metrics, making them suitable for different genomic contexts and study designs.

### Deep learning and convolutional neural networks

DeepVariant distinguishes itself by applying a convolutional neural network (CNN) model, which treats sequencing reads as images. This unique approach allows it to excel in precision (0.7869 for chr20) and recall (0.9864), leading to the highest F1-score (0.8754) among the algorithms tested on chr20. The CNN model in DeepVariant processes sequence data as visual patterns, capturing complex error correlations that traditional statistical models might miss [13]. This image-based model is highly effective for reducing false positives, as reflected by its leading precision score, and contributes to its general robustness across different sequencing technologies. However, despite its high precision, DeepVariant’s reliance on a pre-trained deep learning model can present limitations in adaptability. A key limitation of DeepVariant is its “black-box” nature, which refers to the complexity of its decision-making process. Deep learning models, including CNNs used by DeepVariant, rely on complex, multi-layered feature extraction and decision-making processes that are not easily interpretable. As a result, researchers cannot directly trace or understand how the model reaches its conclusions about specific variants. For instance, the model may classify a region as harboring a true variant or dismiss it as an artifact, but the reasoning behind these classifications is embedded in millions of model parameters and is not accessible to the user [13]. This lack of interpretability can be particularly problematic in research contexts that require transparency, such as clinical genomics or regulatory environments, where the justification for each variant call must be clearly understood and validated. While DeepVariant’s CNN model is highly accurate, it requires substantial computational resources, which may not be feasible for all research settings, especially for whole-genome data where it has not been assessed in this study.

### Probabilistic models and likelihood calculations

Samtools is a widely used tool in variant calling that relies on probabilistic models, specifically genotype likelihood calculations, to infer genetic variants. Unlike more complex Bayesian or machine-learning-based callers, these tools employ straightforward statistical assumptions, including the Hardy-Weinberg Equilibrium (HWE), which posits that allele and genotype frequencies remain constant in a population over generations in the absence of external forces [22]. By adhering to such fundamental principles, Samtools achieves a practical balance between recall and precision, although this comes at the expense of achieving the highest possible precision or recall. The core of this variant calling algorithm is the genotype likelihood calculation, which assesses the probability of the observed sequencing data given each possible genotype at a particular locus. This step is crucial because sequencing data inherently contains noise [22]. Genotype likelihoods help to account for this noise by providing a quantitative measure of how likely each genotype (homozygous reference, heterozygous, homozygous alternate) is based on the observed bases, their quality scores, and mapping qualities [22]. These scores are used to weight the contribution of each read to the overall genotype likelihood, ensuring that higher-quality data has a stronger influence on the final variant call. By incorporating HWE, the tools can apply probabilistic thresholds that favor expected allele frequencies, particularly in well-characterized populations. This assumption is beneficial in reducing false positives, as the likelihoods of observing certain genotypes can be penalized if they significantly deviate from the expected HWE frequencies [23]. However, this approach can be limiting in cases where the population deviates from HWE, such as in populations with a high degree of genetic substructure or in cases where rare variants are more common due to selective pressures. In such cases, the HWE assumption may cause Samtools to overlook low-frequency or population-specific variants, resulting in slightly lower recall compared to tools that employ more adaptive models, such as Bayesian or machine-learning-based approaches. Beyond genotype likelihood calculations, Samtools applies a series of quality thresholds and probabilistic filters to refine variant calls. These include depth of coverage (DP), which ensures that only positions with sufficient read coverage are considered for variant calling, as higher coverage provides more reliable support for variant detection and reduces the likelihood of false positives due to random sequencing errors. Base quality (BQ) is also considered, where bases with low Phred scores are either disregarded or downweighted in the likelihood calculations, minimizing the impact of potentially detecting erroneous reads. Another key metric is strand bias, which is monitored to detect whether variant-supporting reads are disproportionately aligned to only one DNA strand, a possible sign of alignment artifacts; Samtools applies a strand bias filter to reduce the confidence of such variants unless they are observed on both strands. Finally, mapping quality (MQ) scores are used to evaluate the confidence of read alignment to the reference genome. Reads with low MQ scores, which are more likely to be incorrectly aligned (especially in repetitive or complex genomic regions), are downweighted to improve variant calling accuracy. By integrating these filters, Samtools enhances its precision, focusing on high-confidence calls and minimizing false positives. However, this conservative approach also means that variants with low coverage, low quality scores, or other atypical characteristics may be filtered out, potentially leading to missed calls in regions with low complexity or low coverage. The conservative nature of Samtools is evident in its performance metrics: Samtools achieves a precision of 0.6761 and recall of 0.9545 on chr20, and a slightly improved precision (0.7722) and recall (0.9754) on WGS data. These metrics reflect a deliberate design choice to reduce the proportion of false positives through stringent statistical thresholds and quality filters. Samtools is particularly effective at detecting common variants with strong supporting evidence, which makes it ideal for applications where high precision is required and the rate of false positives needs to be minimized. However, this focus on precision comes at the cost of reduced recall in certain cases. In low-complexity or low-coverage regions, Samtools may struggle to detect rare or low-frequency variants, which typically require more nuanced probability modeling to distinguish from sequencing noise. The reliance on HWE and quality thresholds means that the tool is less adaptive to such variants, as it is more likely to filter out signals that do not fit its expected patterns of high-quality, well-supported reads. This contrasts with Bayesian or machine-learning-based tools, which can use priors or learned patterns to increase recall for rare variants without significantly increasing the false positive rate.

### Post-calling filtering

Strelka2 and Varscan2 enhance variant calling through post-calling filtering and statistical tests. However, they differ significantly in their methodologies, with Strelka2 incorporating machine learning to refine its variant calls, while Varscan2 relies more on heuristic filtering and statistical tests for somatic mutation detection. These approaches influence their precision, recall, and F1-score metrics, as well as their suitability for different types of genomic analyses.

#### Strelka2: Machine learning based filtering with random forest.

Strelka2 is designed for both germline and somatic variant detection and employs a likelihood-based model for variant calling, followed by a sophisticated machine learning-based filtering step. After identifying potential variants, Strelka2 uses a random forest classifier in its final filtration step. The random forest model, a type of ensemble machine learning method, consists of multiple decision trees that collectively evaluate a range of variant characteristics to classify each variant as high-confidence or low-confidence. In the random forest model, Strelka2 considers several features of each variant, such as the confidence that a read is correctly aligned to the reference genome (where higher mapping quality scores indicate more reliable alignments and, consequently, more reliable variant calls). Strelka2 also considers the genomic context around each variant, including whether the region is repetitive or has complex sequence structures that may increase the likelihood of alignment errors, and to handle indel error rates, it applies a binomial mixture model to estimate insertion and deletion error rates, which helps improve the precision of indel calling, especially in noisy regions. By combining these features in a random forest classifier, Strelka2 can assign each variant a probability score representing its confidence level. This machine learning model is particularly effective in reducing false positives by filtering out variants that have characteristics typically associated with sequencing artifacts or errors. The random forest model’s ability to adapt to the specific attributes of each dataset helps Strelka2 achieve high precision (0.8326 in WGS) while maintaining a strong recall (0.9813), resulting in an overall F1-score of 0.9009. This combination of high precision and recall makes Strelka2 highly effective at calling variants, particularly insertions and deletions, in complex regions where traditional filtering methods may struggle. The strength of Strelka2’s random forest classifier lies in its flexibility and adaptability. The model can effectively adjust to variations in sequence quality, strand bias, and genomic context, which allows Strelka2 to maintain high precision even in challenging genomic regions. Its ability to handle multiple variables simultaneously makes it well-suited to reduce false positives while retaining true positives, particularly in data with significant noise.

#### Varscan2: Statistical and heuristic filtering.

Varscan2 takes a more traditional approach to variant calling, employing heuristic filtering methods along with statistical tests to classify variants, particularly in tumor-normal pair analyses [25]. Varscan2 is well-suited for somatic mutation detection due to its capability to analyze matched tumor and normal samples simultaneously, which allows it to differentiate between germline and somatic mutations. One of the key components of Varscan2’s variant detection process is Fisher’s exact test, which is used to assess the significance of allele frequency differences between tumor and normal samples. Fisher’s exact test calculates a p-value based on the observed read counts for each allele in the tumor versus normal samples, helping Varscan2 determine whether a variant is likely somatic (specific to the tumor) or germline (present in both the tumor and normal samples). This test is particularly effective for identifying somatic mutations in cancer genomics, where somatic mutations appear at varying frequencies depending on tumor heterogeneity [25]. Varscan2 applies several additional filtering criteria to improve the quality of its variant calls, such as considering strand bias, discarding variants that are predominantly supported by reads from only one DNA strand, variant allele frequency, the position of each supporting read, and read depth. High strand bias often indicates sequencing artifacts rather than true variants. Varscan2 evaluates the position of each supporting read within the read itself; variants that are supported primarily by bases at the ends of reads are more likely to be false positives, as these positions are more prone to sequencing errors. For each variant, Varscan2 calculates the variant allele frequency, which is the proportion of reads supporting the variant allele relative to the total read count at that position. In somatic mutation detection, Varscan2 considers variants with significantly different allele frequencies between tumor and normal samples to be potential somatic mutations. Varscan2 requires a minimum read depth at each variant position, ensuring that low-coverage regions, where sequencing noise may obscure true variants, are not called. While these heuristic filters and statistical tests make Varscan2 effective at identifying somatic mutations, they are generally more rigid than Strelka2’s machine learning model, which dynamically adapts to the data. As a result, Varscan2 has a more limited ability to adjust to complex sequence contexts, resulting in lower precision (0.7220 in chr20) and recall (0.9322) compared to Strelka2. Varscan2’s reliance on fixed thresholds and binary filters can be beneficial for high-confidence variant calling in straightforward cases but may lead to reduced recall in more complex regions with high GC content or in regions with lower coverage, where a more flexible model might be advantageous. In terms of performance, Strelka2 achieves higher precision than Varscan2, particularly for indels and complex genomic regions, due to its random forest classifier, which fine-tunes variant calling based on multiple data features [25]. This approach is especially beneficial in scenarios where variant context, quality, and noise levels vary significantly, such as in whole-genome sequencing (WGS) or cancer genomics. Strelka2’s ability to adapt to these variations results in its high F1-score (0.9009 in WGS), making it a preferred choice for applications requiring a high degree of both precision and recall. Varscan2, in contrast, offers a robust but more rigid approach, suited to studies where high-confidence somatic mutations are the focus. Its use of Fisher’s exact test for comparing tumor and normal samples is particularly effective in detecting somatic mutations, providing a statistical basis for variant calling that can effectively filter out germline variants [25]. However, Varscan2’s reliance on heuristic filters limits its adaptability, which may lead to reduced performance in complex or high-GC regions and slightly lower recall. Its F1-score of 0.8137 indicates that while it is effective for somatic mutation detection, it may not capture the full range of variants in challenging regions or at lower frequencies.

### Performance variability and choosing the right caller

The algorithms show different performance trends between the chr20 subset and WGS data, highlighting the impact of the scope of the dataset and the depth of sequencing on the call for variants. For example, GATK and Strelka2 maintain strong recall in both datasets, indicating consistent performance for both small genomic subsets and full genomes. Octopus also demonstrates high recall in the WGS data (0.9838), likely due to its robust haplotype modeling, which captures true variants even in complex regions. FreeBayes, while effective in capturing a broad range of variants, shows a noticeable increase in precision from chr20 (0.6286) to WGS (0.7394), possibly reflecting better error correction in larger datasets due to the increased data points available for statistical modeling.

**Fig 7:**
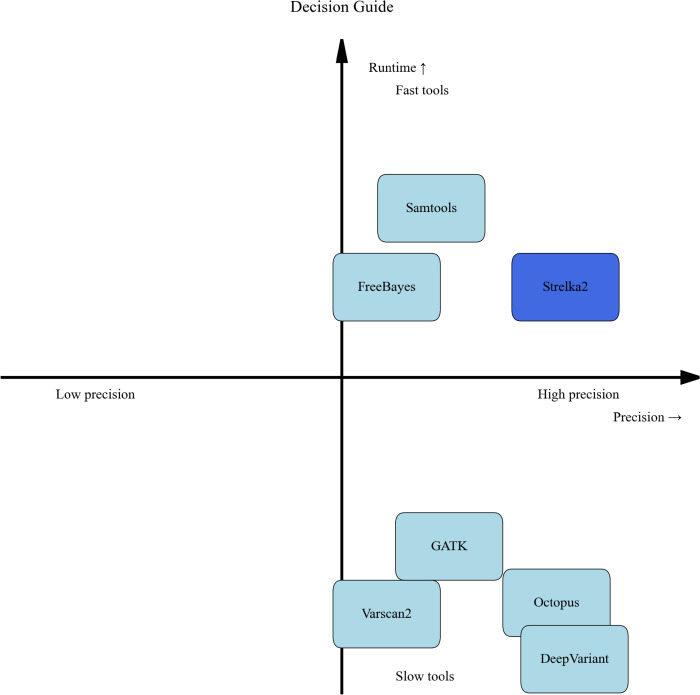
Variant caller selection guide based on precision and runtime performance. This decision guide provides a clear, evidence-based framework for selecting variant callers by visually mapping their performance along two critical operational dimensions: precision and computational runtime.

The decision guide provided in Fig 7 provides a clear, evidence-based framework for selecting variant callers by visually mapping their performance along two critical dimensions: precision and computational efficiency. The spatial distribution of tools reveals fundamental algorithmic trade-offs that directly inform selection strategy. Tools positioned in the upper right quadrant, such as Strelka2, represent the optimal balance of high precision and reasonable runtime, making them ideal for production environments where accuracy and efficiency are both priorities. Conversely, tools like DeepVariant and Octopus, located in the high-precision but slower region, offer superior accuracy at the cost of substantial computational resources—a worthwhile trade-off for clinical applications where precision is paramount. The visualization clearly demonstrates that no single tool excels in all dimensions, emphasizing that selection must be driven by specific research constraints and objectives. This evidence-based approach empowers researchers to make informed decisions that align computational investments with analytical requirements, ultimately enhancing reproducibility and reliability in genomic studies.

In diagnostic pipelines, false-positive variant calls can lead to unnecessary confirmatory testing and potential misinterpretation. Our analysis demonstrates that DeepVariant and Strelka2 achieve the highest precision among evaluated tools, making them particularly suitable for clinical and regulatory-grade applications where accuracy and reproducibility are paramount. By contrast, tools such as FreeBayes, which favor sensitivity, may be better suited to exploratory or research settings where the discovery of novel variants outweighs the risk of occasional false positives.

### Limitations

While this benchmarking highlights DeepVariant’s superior precision, note that the present work does not constitute a clinical validation study. DeepVariant’s suitability for diagnostic applications arises from its demonstrated performance in independent evaluations [12,13], where its high precision and low false-positive rate have been confirmed against well-characterized reference samples. Nonetheless, translation into clinical practice requires independent analytical validation and compliance with accredited laboratory standards such as the U.S. Clinical Laboratory Improvement Amendments [27] and the international Medical laboratories—Requirements for quality and competence [28].

A key limitation is incomplete WGS execution for DeepVariant and VarScan 2 under the specified computational constraints and incomplete execution on chromossome 20 for Strelka 2 due to tool limitations. This limits generalization about these tools at both scenarios within the tested environment. However, published literature indicate DeepVariant and VarScan 2 can complete WGS on higher-performance systems; the present results therefore reflect feasibility under typical lab resources, not absolute capability. The chr20 results remain informative for accuracy profiling, but WGS conclusions are restricted to tools that completed on the defined hardware and budget.

Spearman rank correlations between chr20 and WGS metrics indicate that the relative ranking and overall behavior of the callers are largely preserved from the subset to the full genome. Given the small overlap of callers, statistical testing is not emphasized; interpretation focuses on the direction and magnitude of the associations. These findings support chr20 benchmarking as a representative and lower cost proxy for genome wide performance, while caution is warranted when extrapolating to contexts with atypical coverage or sequence composition, such as highly repetitive or GC rich regions, which may not be fully captured by chromosome 20.

Another limitation of the study is that benchmarking was conducted exclusively on the NA12878 reference genome, which represents a healthy germline sample. While this sample is widely regarded as a gold standard for variant calling performance assessment, its genomic characteristics may not fully capture the complexity of disease-specific or somatic variation contexts, such as tumor heterogeneity, copy-number alterations, or low variant allele frequencies. Consequently, the relative performance of variant callers observed here may differ in cancer or other somatic applications, where distinct error profiles and filtering challenges arise. Future work should therefore extend these comparisons to somatic datasets and clinically relevant scenarios, as demonstrated in recent studies evaluating somatic variant calling performance [5,6].

## Conclusion

This study provides a clear, evidence-based guide for selecting variant callers by comprehensively evaluating seven widely-used tools against a gold-standard benchmark. Our analysis delivers critical insights into the fundamental algorithmic trade-offs that govern performance in genomic variant detection. It is demonstrated that the optimal choice of tool is inherently context-dependent, defined by a balance between accuracy and computational cost. For targeted analyses or smaller datasets, DeepVariant represents the state-of-the-art, achieving the highest precision and F1-score by leveraging its deep learning architecture to minimize false positives. For large-scale whole-genome analyses, Strelka2 emerges as a superior choice, offering an exceptional balance of superior precision and computational efficiency. Octopus achieves remarkable recall, proving highly effective for sensitive detection in complex genomic regions, though this comes at a significant computational cost. Conversely, FreeBayes prioritizes sensitivity above all else, making it well-suited for exploratory analyses where a higher false positive rate is acceptable. Finally, GATK and SAMtools provide robust, balanced performance, with GATK excelling in complex region precision and SAMtools offering a lightweight and efficient solution.

These performance characteristics are direct consequences of underlying algorithmic methodologies, highlighting a central trade-off: increased computational complexity and resource demands are often necessary to achieve gains in precision and accuracy in challenging genomic contexts.

Consequently, we recommend that selection be driven by specific research priorities:

- For clinical or diagnostic settings requiring maximal accuracy: DeepVariant or Strelka2.
- For large-scale cohort studies prioritizing efficiency: Strelka2 or SAMtools.
- For research sensitivity in complex regions: Octopus.
- For exploratory discovery where cost is a constraint: FreeBayes.

By elucidating these performance landscapes and their algorithmic foundations, this work empowers researchers and clinicians to make informed, strategic decisions that optimize workflows, enhance reproducibility, and ultimately, strengthen the validity of genomic findings across diverse applications.

## Supporting information

S1 Table Variant calling characteristics computed on NA12878 subset (chr20) using RTG Tools. Ordered by number of SNPs detected.
(PDF)

S2 Table Variant calling characteristics computed on NA12878 WGS using RTG Tools. Ordered by number of SNPs detected.
(PDF)

S3 Table Performance metrics on the NA12878 subset (chr20). Ordered by F1-score.
(PDF)

S4 Table Performance metrics on NA12878 whole-genome sequencing (WGS) data. Ordered by F1-score.
(PDF)

S1 File Illumina Resequencing Report Summary for NA12878. Comprehensive quality metrics and variant calling results for the NA12878 reference sample sequenced on the Illumina HiSeq 2000 platform. The report details: (1) Sample information; (2) Alignment statistics demonstrating high mapping efficiency; (3) Uniform coverage distribution; (4) Small variant summary; (5) Structural variant calls; (6) Fragment length metrics; and (7) Analysis specifications using Isaac aligner (v04.16.09.24) and GRCh38 reference genome. This report establishes the high data quality foundation for subsequent variant caller benchmarking.
(PDF)
