# Supplementary material for: Variant calling in genomics: A comparative performance analysis and decision guide
Source: PLoS One. 2026 Feb 5;21(2):e0339891. doi: 10.1371/journal.pone.0339891 (PMC12875585; doi:10.1371/journal.pone.0339891)
Supplement: S1 Table — Ordered by number of SNPs detected. (PDF) [file pone.0339891.s001.pdf]

S1 Table. Variant Calling Statistics Across Different Tools

**Variant Calling Statistics Across Different Tools**						
**Statistics**	**GATK**	**Samtools**	**FreeBayes**	**Varscan2**	**Octopus**	**DeepVariant**
Failed Filters	0	0	0	2,896	9,080	59,970
Passed Filters	123,220	116,952	328,360	62,770,558	111,002	103,883
SNPs	103,825	101,639	100,911	93,615	89,832	85,840
Insertions	9,201	7,779	7,687	6,597	10,323	8,706
Deletions	9,593	7,367	8,624	6,750	9,282	8,977
Indels	275	167	2,504	0	88	346
Same as reference	0	0	202,694	61,718,239	0	14
SNP Ti/Tv	1.92	1.93	1.88	2.03	2.07	2.00
Total Het/Hom ratio	2.50	2.13	2.39	2.37	2.07	1.77
SNP Het/Hom ratio	2.52	2.27	2.34	2.20	2.04	1.70
Insertion Het/Hom ratio	2.06	1.35	2.27	3.72	1.92	2.09
Deletion Het/Hom ratio	2.52	1.54	2.18	5.09	2.04	2.15
Indel Het/Hom ratio	-	-	2.59	-	7.80	-
Insertion/Deletion ratio	0.96	1.06	0.89	0.98	1.11	0.97

Comparison of variant calling statistics across six different variant callers (GATK, Samtools, FreeBayes, Varscan2, Octopus, and DeepVariant) applied to the same genomic dataset. Values represent counts of variants or calculated ratios as indicated in the first column.
