# Supplementary material for: Variant calling in genomics: A comparative performance analysis and decision guide
Source: PLoS One. 2026 Feb 5;21(2):e0339891. doi: 10.1371/journal.pone.0339891 (PMC12875585; doi:10.1371/journal.pone.0339891)
Supplement: S2 Table — Ordered by number of SNPs detected. (PDF) [file pone.0339891.s002.pdf]

S2 Table. Variant Calling Statistics Across Different Tools (Sample Comparison)

**Variant Calling Statistics Across Different Tools (Sample Comparison)**					
**Samples**	**Samtools**	**GATK**	**FreeBayes**	**Octopus**	**STRELKA2**
Failed Filters	0	0	0	284,329	488,555
Passed Filters	4,920,844	4,930,670	12,560,020	4,823,270	4,590,257
SNPs	4,119,113	4,084,918	4,016,387	3,856,679	3,796,849
MNPs	0	0	171,642	173	0
Insertions	396,584	399,552	339,970	464,673	383,869
Deletions	391,684	418,836	385,053	425,018	395,737
Indels	13,463	13,627	103,179	3,396	13,802
Same as reference	0	0	7,543,789	0	0
SNP Ti/Tv	1.93	1.95	1.95	2.02	2.02
Total Het/Hom ratio	1.60	1.71	1.71	1.60	1.57
SNP Het/Hom ratio	1.58	1.68	1.66	1.53	1.55
Insertion Het/Hom ratio	1.59	1.69	1.93	1.69	1.58
Deletion Het/Hom ratio	1.81	1.89	1.72	1.65	1.75
Indel Het/Hom ratio	-	-	2.03	2.95	-
Insertion/Deletion ratio	1.01	0.95	0.88	1.09	0.97

Comparison of variant calling statistics across five different variant callers (Samtools, GATK, FreeBayes, Octopus, and STRELKA2) applied to multiple samples. Values represent counts of variants or calculated ratios as indicated in the first column. MNPs refer to Multi-Nucleotide Polymorphisms.
