# Supplementary material for: Variant calling in genomics: A comparative performance analysis and decision guide
Source: PLoS One. 2026 Feb 5;21(2):e0339891. doi: 10.1371/journal.pone.0339891 (PMC12875585; doi:10.1371/journal.pone.0339891)
Supplement: S3 Table — Ordered by F1-score. (PDF) [file pone.0339891.s003.pdf]

S3 Table. Performance Metrics of Variant Callers

**Performance Metrics of Variant Callers**			
**Variant Caller**	**Precision**	**Recall**	**F1-score**
DeepVariant	**0.7869**	**0.9864**	**0.8754**
Octopus	0.7412	0.9862	0.8463
Varscan2	0.7220	0.9322	0.8137
GATK	0.6617	0.9847	0.7915
Samtools	0.6761	0.9545	0.7915
FreeBayes	0.6286	0.9758	0.7646

Comparison of performance metrics (Precision, Recall, and F1-score) across six variant callers. Bold values indicate the highest score in each column, with DeepVariant achieving the best performance across all three metrics.
