# Supplementary material for: Variant calling in genomics: A comparative performance analysis and decision guide
Source: PLoS One. 2026 Feb 5;21(2):e0339891. doi: 10.1371/journal.pone.0339891 (PMC12875585; doi:10.1371/journal.pone.0339891)
Supplement: S4 Table — Ordered by F1-score. (PDF) [file pone.0339891.s004.pdf]

S4 Table. Performance Metrics of Variant Callers

**Performance Metrics of Variant Callers**			
**Variant Caller**	**Precision**	**Recall**	**F1-score**
Octopus	0.8005	**0.9838**	**0.8827**
STRELKA2	**0.8326**	0.9813	0.9009
GATK	0.7766	**0.9837**	0.8680
Samtools	0.7722	0.9754	0.8620
FreeBayes	0.7394	0.9748	0.8409

Comparison of performance metrics (Precision, Recall, and F1-score) across five variant callers. Bold values indicate the highest score in each column. STRELKA2 achieved the highest precision, while Octopus and GATK tied for the highest recall, and Octopus achieved the highest F1-score.
