# Supplementary material for: Variant calling in genomics: A comparative performance analysis and decision guide
Source: PLoS One. 2026 Feb 5;21(2):e0339891. doi: 10.1371/journal.pone.0339891 (PMC12875585; doi:10.1371/journal.pone.0339891)
Supplement: S1 File — Comprehensive quality metrics and variant calling results for the NA12878 reference sample sequenced on the Illumina HiSeq 2000 platform. The report details: (1) Sample information; (2) Alignment statistics demonstrating high mapping efficiency; (3) Uniform coverage distribution; (4) Small variant summary; (5) Structural variant calls; (6) Fragment length metrics; and (7) Analysis specifications using Isaac aligner (v04.16.09.24) and GRCh38 reference genome. This report establishes the high data quality foundation for subsequent variant caller benchmarking. (PDF) [file pone.0339891.s005.pdf]

Resequencing Report

Sample: NA12878

Workflow: Resequencing

Report Date: 2017-08-24T13:53:07

© 2017 Illumina, Inc. All rights reserved.

For Research Use Only. Not for use in diagnostic procedures.

Sample Information

Sample ID	Total PF Reads	Percent Q30 Bases
NA12878	1,586,092,978	92.33%

Read Level Statistics

Read	Total Aligned Reads	Percent Aligned Reads
1	764,564,883	96.41%
2	762,505,637	96.15%

Base Level Statistics

Read	Percent Q30 Bases	Total Aligned Bases	Percent Aligned Bases	Percent Mismatches
1	93.42%	75,012,148,175	94.59%	0.25%
2	91.23%	74,727,019,630	94.41%	0.38%

© 2017 Illumina, Inc. All rights reserved.

For Research Use Only. Not for use in diagnostic procedures.

Coverage Histogram (mean coverage 50.08)

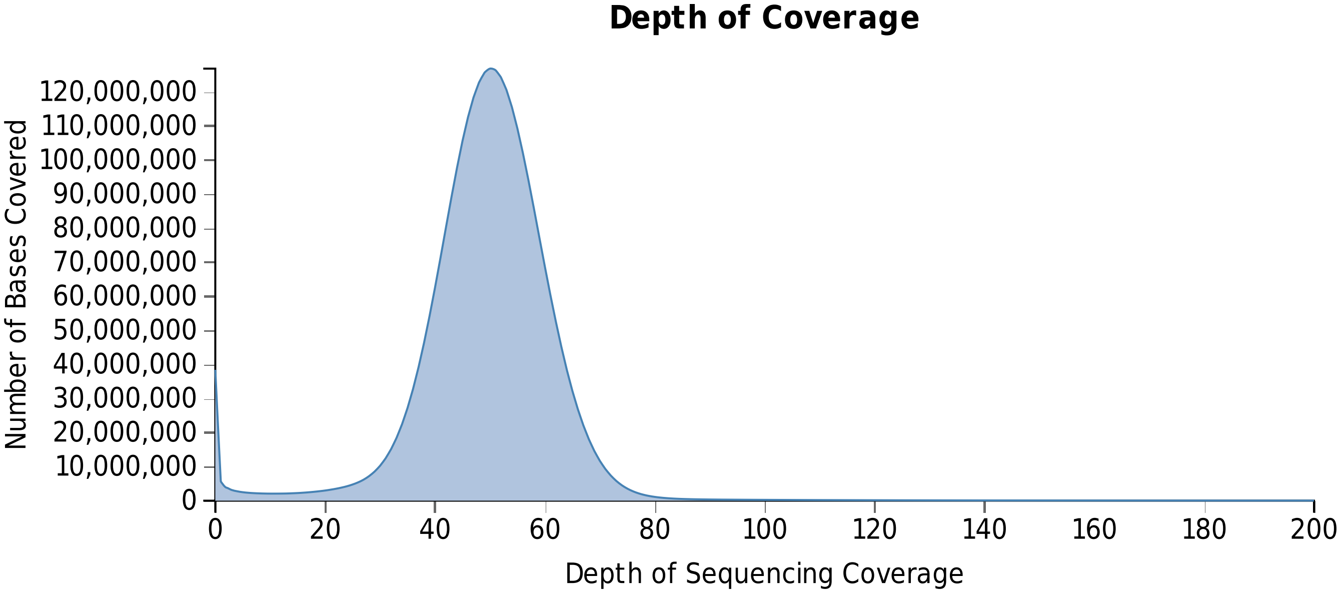

© 2017 Illumina, Inc. All rights reserved.

For Research Use Only. Not for use in diagnostic procedures.

Small Variants Summary

	SNVs	Insertions	Deletions
Total Passing	3,782,261	362,705	383,759
Percent Found in dbSNP	97.93%	92.05%	92.72%
Het/Hom Ratio	1.568	1.840	2.053
Ts/Tv Ratio	2.036	-	-

Variants by Sequence Context

	SNVs	Insertions	Deletions
In Genes	1,780,449	178,076	189,196
In Exons	55,339	4,057	4,076
In Coding Regions	22,668	241	255
In UTR Regions	32,671	3,816	3,821
In Splice Site Regions	3,366	312	357
In Mature microRNA	0	0	0

Genes include exons, introns and UTR regions. Exons include coding and UTR regions. UTR regions include 5' and 3' UTR regions. Splice site regions include regions annotated as splice acceptor, splice donor, splice site or splice region.

Variants by Consequence

	SNVs	Insertions	Deletions
Frameshifts	-	66	77
Non-synonymous	10,799	172	174
Synonymous	11,776	-	-
Stop Gained	76	5	0
Stop Lost	13	0	0

Variation consequences are calculated following the guidelines at
http://uswest.ensembl.org/info/genome/variation/predicted_data.html#consequences

© 2017 Illumina, Inc. All rights reserved.

For Research Use Only. Not for use in diagnostic procedures.

Structural Variants Summary

Variant Type	Total	In Genes
CNVs	326	173
SV Insertions	1,536	773
SV Deletions	7,183	2,631
SV Tandem Duplications	586	236
SV Inversions	952	563
SV Breakends	2,162	558

© 2017 Illumina, Inc. All rights reserved.

For Research Use Only. Not for use in diagnostic procedures.

Fragment Length Summary

Fragment Length Median	Minimum	Maximum	Standard Deviation
319 bp	89 bp	1282 bp	75 bp

Note: The minimum and maximum are calculated from values within approximately three standard deviations (excluding the lower and upper 0.15% of the data) to account for potential outliers.

Duplicate Information

Percent Duplicate Proper Read Pairs
1.05%

© 2017 Illumina, Inc. All rights reserved.

For Research Use Only. Not for use in diagnostic procedures.

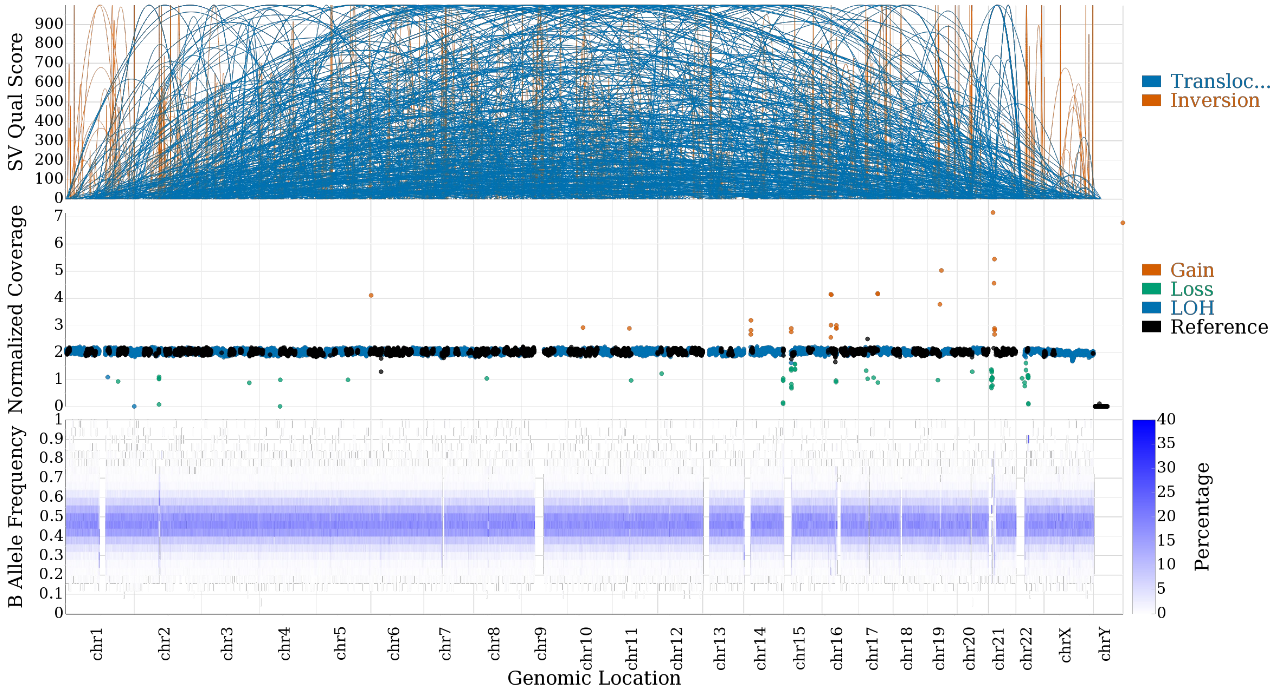

© 2017 Illumina, Inc. All rights reserved.

For Research Use Only. Not for use in diagnostic procedures.

Analysis Details

Settings

Setting Name	Value
Run Folder	RunFolder
Reference Genome	Homo Sapiens (NCBI GRCh38 with decoys)
Annotation Source	Ensembl
Flag PCR Duplicates	True
SV Caller	Manta
CNV Caller	Canvas

Software Versions

Software	Version
SAMtools	1.2
Isaac (Aligner)	Isaac-04.16.09.24
Strelka Germline Variant Caller	2.7.1
Manta (SV Caller)	1.0.2
Canvas (CNV Caller)	1.19.1
Illumina Annotation Engine	1.5.2.0
Pluggable Universal Metrics Analyzer (PUMA)	00.15.11.02
PUMA Metrics	1.0.16.1
Whole Genome Sequencing (BaseSpace Workflow)	6.0.0.0
Resequencing Workflow	5.11.0
HLA Typer	5.11.0
Expansion Hunter	2.0.5
Invariant	0.1.5
Consanguinity	2.37.3

Data collections

Data Collection	Version
Annotation Dataset	84.22.36

© 2017 Illumina, Inc. All rights reserved.

For Research Use Only. Not for use in diagnostic procedures.
